# Siglec-15 Is an Immune Suppressor and Potential Target for Immunotherapy in the Pre-Metastatic Lymph Node of Colorectal Cancer

**DOI:** 10.3389/fcell.2021.691937

**Published:** 2021-10-13

**Authors:** Hang Du, Jingling Tang, Xiaoyun Li, Xinjun Wang, Liyun Wu, Ruyi Zhang, Pingsheng Hu, Yuan Yang

**Affiliations:** ^1^Clinical Medical Research Center, The Affiliated Hospital of Guizhou Medical University, Guizhou, China; ^2^Department of Anorectal Surgery, The Affiliated Hospital of Guizhou Medical University, Guizhou, China; ^3^Department of Research and Development, Sinorda Biotechnology Co., Ltd, Guizhou, China

**Keywords:** pre-metastatic niche, lymph nodes, colorectal cancer, immunosuppressive, *SIGLEC15*

## Abstract

Lymph node metastasis indicates a poor prognosis in colorectal cancer. To better understand the underlying mechanisms of lymph node metastasis, we analyzed transcriptome characteristics of the pre-metastatic lymph node, a putative microenvironment favorable for the seeding and proliferation of cancer cells. Thus, we tried to compare and elucidate the transcriptional and immune characteristics of sentinel lymph nodes (SNs) with matched non-sentinel lymph nodes (NSNs) in colorectal cancer patients. In this study, a total of 38 pairs of SNs and NSNs were collected, in which 26 pairs of non-metastatic lymph nodes were subjected to RNA-seq and bioinformatics analysis for the gene expression profiles. There were 16 differentially expressed genes between SNs and NSNs being identified, including 9 upregulated and 7 downregulated genes in SN. Gene Ontology (GO) classification analysis revealed that the differentially expressed genes were mainly involved in leukocyte differentiation, chemokine secretion, and immune system regulation. In the meantime, gene set enrichment analysis (GSEA) showed that immune-related signaling pathways, such as transforming growth factor beta (TGF-β) signaling and tumor necrosis factor alpha (TNF-α)/nuclear factor kappa-light-chain-enhancer of activated B cells (NF-κB) signaling, were enriched in NSN, while cell proliferation–related signaling pathways were enriched in SN, including MYC signaling and G2M checkpoint signaling. We further identified *SIGLEC15* as a top upregulated gene in SN. However, RNAscope assay showed that *SIGLEC15* was not largely co-expressed with M2 macrophage marker *CD163*. We then selected eight pairs of lymph nodes for further cytological studies. Flow cytometry analysis revealed that Siglec-15 was expressed on all myeloid cell subsets. The relative expression of *SEGLEC15* (SN/NSN) was correlated with the microsatellite instability (MSI) status in colorectal cancer patients. Further studies found that small interfering ribonucleic acid (siRNA)-mediated silencing of *SLGLEC15* can enhance the anti-tumor function of T cells, as indicated by cytokine release analysis. In conclusion, we presented here a first report on the gene expression profiling of the pre-metastatic lymph node in colorectal cancer. The findings in this study suggest that *SIGLEC15* plays an important role in SN immunosuppression. *SEGLEC15* silencing could be a therapeutic strategy for restoring T cell function in tumor SNs.

## Introduction

In colorectal cancer (CRC), distant metastasis is the leading cause of death (Bray et al., 2018). Lymph node is considered as the main venue of distant metastasis (Ulintz et al., 2018), and the lymph node metastasis usually indicates a poor prognosis (Gunderson et al., 2010). Therefore, deeper insights into the underlying mechanisms of lymph node metastasis will benefit the CRC treatment.

While plenty of studies on colorectal tumorigenesis have been carried out over the years (Church, 2016), relatively few studies were focused on the changes having occurred in the lymph node. It has been shown that gene expression alterations in tumor were associated with lymph node metastasis. In these cases, several factors were related to lymph node metastasis and poor prognosis in CRC, including *NOTCH3* (Varga et al., 2020), *FANCD2* (Ozawa et al., 2010), *FOXM1* (Li et al., 2013), *TFF3* (Yusup et al., 2017), and *UBN2* (Zhao et al., 2019). Likewise, DNA copy number variation in tumor may be linked to lymph node metastasis. For instance, *RASAL2* (Pan et al., 2018) gain and *FAM134B* (Kasem et al., 2014) loss were found to be associated with poor prognosis and lymph node metastasis in CRC. In addition, changes in microRNA (miRNA) (Fujino et al., 2017), long non-coding RNA (lncRNA) (Shen et al., 2017), circular RNA (CircRNA) (Lu et al., 2020), and gene expression pattern (Watanabe et al., 2009) have also been proven to be associated with lymph node metastasis. However, almost all of these studies were only directed at the tumor itself rather than lymph nodes.

The pre-metastatic niche provides a microenvironment for tumor cell seeding and proliferation. Studies on the lymph node pre-metastatic niche could improve our understanding of how tumor metastasis happens. Multiple studies have identified certain features of the pre-metastatic lymph node, including immunological alterations (Kim et al., 2006), lymph angiogenesis (Harrell et al., 2007), high endothelial venule remodeling (Chung et al., 2012), increase of immunosuppressive cells [such as Tregs, myeloid-derived suppressor cells (MDSCs), and tumor-associated macrophage (TAMs)] (Deng et al., 2010; Ogawa et al., 2014), and decreased number of effector lymphocytes or lymphocyte dysfunction (Ito et al., 2006). To date, the underlying molecular mechanisms of the lymph node pre-metastatic niche, especially in colorectal cancer, remain elusive.

In this study, we defined the pre-metastatic lymph node as the sentinel lymph node (SN) without tumor invasion. The aim of this study was to characterize alterations taking place in the SNs on the gene and cellular levels. This study may facilitate our understanding of changes in the lymph node microenvironment prior to tumor cell metastasis and provide a potential strategy for preventing lymph node metastasis in colorectal cancer.

## Materials and Methods

### Patients and Specimens

A total of 38 pairs of SN and non-sentinel lymph node (NSN) were collected from CRC patients who underwent surgery between October 2018 and June 2020 in the Department of Anorectal Surgery at the Affiliated Hospital of Guizhou Medical University. Ethical approval was granted by the Medical Ethics Committee of the Affiliated Hospital of Guizhou Medical University (approval numbers 2014-40 and 2018-057). All patients have signed a written informed consent. For each patient, 1 ml of patent blue V tracer was injected into the subserosa at four points around the tumor within 5 min after the removal of tumor tissue. Blue-stained lymph nodes within the first 3 min following dye application were marked with a suture and collected *ex vivo* as SNs, while lymph nodes with negative blue staining near the tumor were defined as NSN.

The lymph nodes were excised and cut in half; one-half was retained for RNA-seq or cytological examination, and the remaining half was processed for routine histopathological studies. Immunohistochemistry staining was performed to detect the expression of Pan Cytokeratin (pan-CK) (AE1 + AE3) (1:200, Abcam) in paired lymph nodes (Supplementary Figure 1A). In this study, 38 pairs of lymph nodes comprised 4 pairs of metastatic lymph nodes and 34 pairs of non-metastatic lymph nodes. Among the 34 pairs of non-metastatic lymph nodes, there were 26 pairs randomly selected for RNA-seq, and the remaining 8 were subjected to cytological examination. The flow chart of sample processing was illustrated in Supplementary Figure 1B.

### RNA Sequencing

RNA-Seq experiments were performed by Novogene (Beijing, China). RNA-seq analysis was carried out using TRIzol Reagent (Invitrogen). Libraries were indexed and sequenced using NOVO6000 (Illumina) platform. The quality control (QC) of base qualities and nucleotide composition of sequences were performed by FastQC to identify problems in library preparation or sequencing. The sequence quality for the dataset described here was sufficient that no reads were trimmed or filtered prior to the alignment analysis. Paired-end reads were aligned to the human genome (hg19) using the Hisat2 program (v2.0.5). Feature Counts v1.5.0-p3 was used to count the reads numbers mapped to each gene, and the counts were then loaded into R package DESeq2 to identify differentially expressed genes (DEGs) with a cut-off as adjusted *p*-value < 0.05.

### Gene Ontology Classification Analysis

In order to determine the putative functions of the DEGs, Gene Ontology (GO) classification into GO-BP (biological process), GO-MF (molecular function), and GO-CC (cellular component) was carried out by using clusterProfiler R package (v3.14.3). *P*-value < 0.05 was set as the cut-off criterion for the significant enrichment.

### Gene Set Enrichment Analysis

Gene set enrichment analysis (GSEA) was performed to investigate the biological characteristics that are significantly different between SN and NSN. GSEA analysis was conducted by using Pi:xPierGSEA R package (v1.14.0) based on H (hallmark gene sets) from the Molecular Signatures Database (MSigDB v7.1.1). GSEA was first applied to the ranking that was defined by the log2 fold change (log_2_FC) of the differential expression analysis using DESeq2. The entire ranked list was then used to calculate the enrichment score for each gene set, assessing how the genes of each gene set are distributed across the ranked list. The normalized enriched score (NES) was determined for each gene set. The significant enrichment of gene set was identified based on the absolute values of NES > 1, *p*-value ≤ 0.05, and false positive rate (FDR) ≤ 0.05.

### Reverse Transcription Polymerase Chain Reaction-Based Validation of *SIGLEC15* Expression

Reverse transcription polymerase chain reaction (RT-PCR) was conducted to validate the expression of *SIGLEC15*. Total RNA was extracted by using TRIzol reagent following the manufacturer’s instructions. cDNA synthesis was performed using the PrimeScript^TM^ RT reagent Kit with gDNA Eraser (Takara, Japan). The following primers were designed using Primer5 software: *SIGLEC15* forward 5′-GCCACCTAGTGACCGCCGAACT-3′, reverse 5′-CAGCGCCTTGAAGCCGAGA-3′; *ACTB* forward 5′-TGACGTGGACATCCGCAAAG-3′, reverse 5′-CTGGAAGGT GGACAGCGAGG-3′. RT-PCR was performed in a 96-well plate on an ABI VIIA7 Dx (Applied Biosystems, Foster City, CA, United States) using TB Green^®^ Premix Ex Taq^TM^ II (Takara, Japan) according to the manufacturer’s instructions. Each biological replicate was tested in triplicate. The relative expression values of *SIGLEC15* were calculated using the 2^–ΔΔ*Ct*^ method and normalized against the expression levels of *ACTB* gene.

### RNAscope *in situ* Hybridization

Paraffin-embedded lymph node sections (4 μm) were processed for RNA *in situ* detection using the RNAscope^®^ 2.5 HD Duplex Reagent Kit according to the manufacturer’s instructions (Advanced Cell Diagnostics, Newark, CA, United States). The following RNAscope probes were used: *CD163* (NM_203416.3, region 210-1565) and *SIGLEC15* (NM_213602.2, region 2-1011). Immunostained slides were scanned using the Aperio Scanscope XT high-resolution scanner at 40× magnification (Leica Biosystems, Mt. Waverley, VIC, Australia). The positive signal was quantitatively analyzed by StrataQuest v6 software (TissueGnostics, Vienna, Austria).

### Flow Cytometry

The expression of Siglec-15 in various cell subsets was evaluated by flow cytometry. Single-cell suspensions were prepared by enzymatic digestion of lymph nodes with 4% type IV collagenase, 2% hyaluronidase, and 0.4% DNase at 37°C for 60 min. Cells were stained with antibodies (Supplementary Table 1) for 30 min on ice according to the manufacturer’s instructions. Dead cells were removed by using the Zombie Yellow Fixable Viability Kit (BioLegend, San Diego, CA, United States). After incubation, cell suspensions were washed with phosphate-buffered saline (PBS), and the cell pellets were then resuspended in 0.5 ml of PBS for analysis. The following cell subsets were identified by the indicated gating strategies (Supplementary Figure 2): Dendritic cells (DCs) (CD45+CD11b+CD11c+), granulocyte cells (CD45+CD11b+CD15+CD14−), M1 macrophage (CD45+CD11b+CD11c-CD86+), M2 macrophage (CD45+CD11b+CD11c-CD163+), NK cells (CD45+CD3-CD56+), B cells (CD45+CD3-CD19+), T cells (CD45+CD3+), Th cells (CD45+CD3+CD4+), Tc cells (CD45+CD3+CD8+), and monocytes (CD45+CD11b+CD15-CD14+). The mean fluorescence intensity (MFI) of Siglec-15 in different cell subsets was detected and compared between SN and NSN.

### Transient Silencing of *SIGLEC15*

A panel of four *SIGLEC15*-small interfering ribonucleic acids (siRNAs) (S15-96, S15-965, S15-138, and control-siRNA) was designed and synthesized by Genpharma (El Jadida, Morocco). Cell transfection with *SIGLEC15*-siRNA was performed using the AccellTM siRNA Delivery MEdia kit according to the manufacturer’s instructions. Briefly, SN cells (2 × 10^5^ cells/well) were seeded in 96-well plates and gene silencing was conducted in serum-free condition with 20μM siRNA. After 72 h of transfection, cells were harvested for RNA extraction and evaluation of *SIGLEC15* expression by RT-PCR. Flow cytometry was performed to detect the reduced expression of Siglec-15 protein at 96 h after siRNA transfection. The experiment was repeated three times.

### Cytokine Analysis

The secretion of anti-tumor cytokines from SN-derived T cells with *SIGLEC15* knockdown was analyzed using LEGENDplex^TM^ Multi-Analyte Flow Assay Kit (BioLegend, San Diego, CA, United States) as described previously (Wallrapp et al., 2017). Single-cell suspension prepared from the SN of five CRC patients were cultured in serum-free medium and transfected with *SIGLEC15*-specific siRNA or control siRNA for 72 h. The transfected cells were stimulated with phorbol 12-myristate 13-acetate (PMA) and ionomycin overnight, and the production of interleukin-2 (IL-2), Interferon-γ (IFN-γ), and tumor necrosis factor alpha (TNF-α) in the supernatant was determined by flow cytometry.

### Statistical Analysis

Statistical analyses were performed using R 3.6.1 and GraphPad Prism 8.0.2. Comparisons between groups were made by using paired Student’s *t*-test or paired Wilcoxon signed rank test, as appropriate. *P* < 0.05 was considered statistically significant.

## Results

### Patient Characteristics

Thirty-eight CRC patients were enrolled, and the patient characteristics were summarized in Table 1. Pathological examination revealed that 22 patients had no lymph node metastasis (Stages I and II), while 16 patients displayed lymph node metastasis (Stages III and IV). Notably, there was an equal number of patients with left-sided colorectal tumors and those with right-sided colorectal tumors. Microsatellite instability–high (MSI-H) status was identified in six patients.

**TABLE 1:** Clinical characteristics of CRC patients.

Characteristic	N	%
**Age**		
Mean (SD)	63.47 (12.88)	
Range	34–90	
**Gender**		
Male	22	57.89
Female	16	42.11
Tumor location		
Left side	19	50.00
Right side	19	50.00
**T classification (TNM)**		
T2	3	7.89
T3	34	89.47
T4b	1	2.63
**N classification(TNM)**		
N0	23	60.53
N+	15	39.47
**M classification (TNM)**		
M0	34	89.47
M1	4	10.53
**Clinical stage**		
I	3	7.89
II	19	50.00
III	12	31.58
IV	4	10.53
**Microsatellite instability(MSI)**		
MSI-H	6	15.79
MSI-L/MSS	32	84.21

*MSI-H, high-frequency MSI; MSI-L, low-frequency MSI; MSS, microsatellite stable.*

### Genes and Gene Sets Related to the Lymph Node Pre-Metastatic Niche Formation in Colorectal Cancer Patients

**FIGURE 1:**
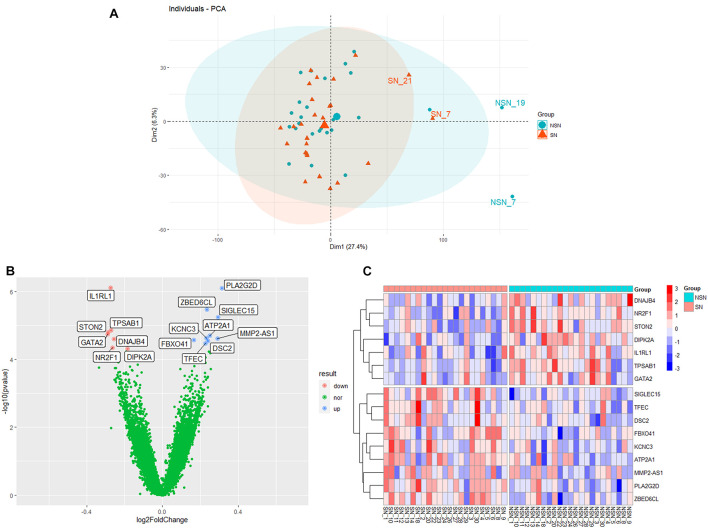
Differential gene expression profiles. **(A)** Principal component analysis (PCA) plot of individual sample. **(B)** Volcano plot of differentially expressed genes (DEGs). **(C)** Clustering heat map of differential gene expression.

We performed RNA-seq on 26 pairs of non-metastatic SN and NSN to examine the gene expression profiles, and principal component analysis led to exclude three outlier pairs of lymph nodes (Figure 1A). As presented in Figure 1B, a total of 16 genes were significantly differentially expressed between SN and the corresponding NSN (adjusted *p*-value < 0.05), including 9 upregulated and 7 downregulated genes. In addition, the sample clustering revealed a distinguishable gene expression pattern between the two groups (Figure 1C).

**FIGURE 2:**
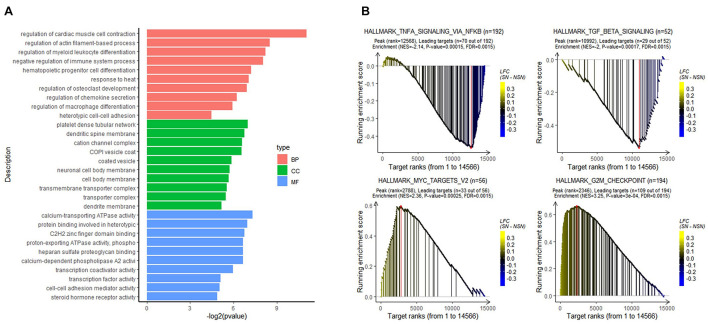
Results of functional enrichment analysis. **(A)** Gene Ontology (GO) analysis of the DEGs. **(B)** gene set enrichment analysis (GSEA) of all expressed genes using the GSEA hallmark pathways database.

To analyze the biological function of the identified DEGs, GO classification was performed. As shown in Figure 2A, the DGEs were mainly enriched in biological processes, such as regulation of myeloid leukocyte differentiation, regulation of chemokine secretion, and regulation of immune system process.

To determine signaling pathways associated with lymph node pre-metastatic niche formation, GSEA analysis was performed on all expressed genes. As illustrated in Figure 2B, while immune-related signaling pathways such as HALLMARK_TGF_BETA_SIGNALING and HALLMARK_TNFA_SIGNALING_VIA_NFKB were highly enriched in the NSN group, signaling pathways related to cell proliferation, e.g., HALLMARK_MYC_TARGETS_V2 and HALLMARK_G2M_CHEKPOINT, were highly enriched in the SN group.

### *SIGLEC15* Expression Is Upregulated in Lymph Node Pre-Metastatic Niche

Transcriptome analysis in this study identified *SIGLEC15* gene as the most upregulated gene in SN. Given that *SIGLEC15* regulates osteoclast differentiation and suppresses T cell responses (Hiruma et al., 2011; Wang et al., 2019), we chose *SIGLEC15* for validating the RNA-Seq data. RT-PCR assay showed that the mRNA expression of *SIGLEC15* was significantly increased in SN compared with NSN among eight pairs of lymph nodes (Supplementary Figure 3).

### *In situ* mRNA Expression of *SIGLEC15* and *CD163*

**FIGURE 3:**
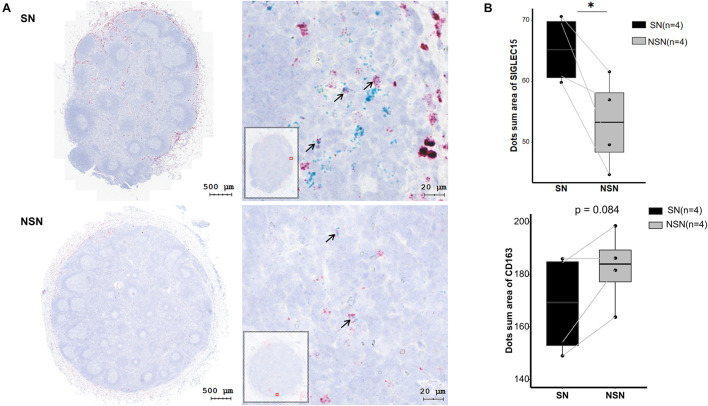
*SIGLEC15* and *CD163* mRNA expression detected by RNAscope. **(A)** Representative images of RNAscope of *SIGLEC15* (green) and *CD163* (red) in sentinel lymph node (SN) and non-sentinel lymph node (NSN). **(B)** Quantification of the expression of *SIGLEC15* and *CD163* mRNA by RNAscope. ^∗^*p* < 0.05, with paired *t*-test.

It has been shown that *SIGLEC15* is predominantly expressed on M2 macrophages (Takamiya et al., 2013). Thus, we investigated the expression of M2 macrophage markers *CD163* and *SIGLEC15* by an RNAscope *in situ* hybridization assay. The data revealed that only a fraction of M2 macrophages co-expressed *SIGLEC15* and *CD163* molecules (Table 2), indicating that *SIGLEC15* is also expressed in other cell types in the lymph nodes. Furthermore, SIGLEC15 displayed as a non-characteristic distribution in the anatomical structure of SN and NSN. In addition, *CD163* expression tended to be concentrated in the subcapsular sinus of the SNs, which is the main area of immune recognition (Louie and Liao, 2019), whereas NSN did not show this feature (Figure 3A). We further quantified the expression of SIGLEC15 and CD163 by StrataQuest v6 software. As depicted in Figure 3B, while higher SIGLEC15 expression was detected in SN as compared to that of NSN, there was no difference in the expression level of CD163 between these two types of lymph nodes.

**TABLE 2:** The co-expression of SIGLEC15 and CD163 in lymph nodes.

Sample ID	SIGLEC15+ cell number	CD163+ cell number	Dual positive cell number	Dual/CD163+ fraction (%)
SN_18	12,865	10,776	1,187	11.02
NSN_18	5,574	6,069	398	6.56
SN_30	16,435	12,226	1,615	13.21
NSN_30	2,292	9,384	337	3.59
SN_14	35,438	45,510	4,101	9.01
NSN_14	13,382	24,710	1,911	7.73
SN_27	4,502	20,950	1,315	6.28
NSN_27	3,304	11,257	366	3.25

### The Expression Pattern of Siglec-15 Protein in Lymph Node Cell Subsets

**FIGURE 4:**
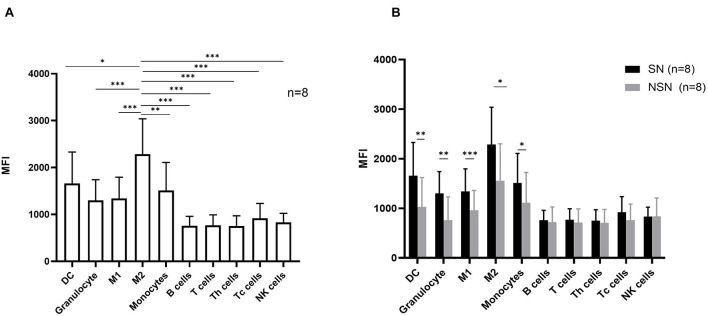
The expression pattern of Siglec-15 protein in lymph node cell subsets. **(A)** The mean fluorescence intensity (MFI) of Siglec-15 in SN cell subsets determined by flow cytometry. *p < 0.05, **p < 0.01, ***p < 0.001, with one-way ANOVA *post-hoc* Dunnett’s. **(B)** Flow cytometry analysis of Siglec-15 expression in respective lymph node subsets. *p < 0.05, **p < 0.01, and ***p < 0.001, with paired *t*-test.

We next investigated the protein expression of Siglec-15 on various cell subsets in both SN and NSN. In SN, myeloid cells including DC cells, M1 macrophages, M2 macrophages, monocytes, and granulocytes had a higher protein level of Siglec-15 than T, B, and NK lymphocytes (Figure 4A). Among myeloid cells, M2 macrophages expressed the highest level of Siglec-15. Comparative studies showed that while a higher expression of Siglec-15 protein was present in all myeloid cell subsets of SN compared with those of NSN, a similar expression level in all lymphocyte subsets was found between SN and NSN (Figure 4B).

### Correlation Between *SIGLEC15* Expression and Clinical Characteristics

**FIGURE 5:**
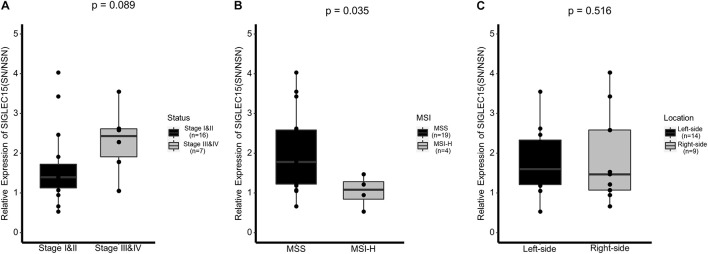
Association of the relative *SIGLEC15* mRNA expression (SN/NSN) and three clinical characteristics. **(A)** Comparison of different clinical stages. **(B)** Comparison of different microsatellite instability (MSI) statuses. **(C)** Comparison of different primary tumor locations. MSS, microsatellite stable; MSI-H, microsatellite instability–high. *p < 0.05, with Wilcoxon test.

Furthermore, we analyzed the correlation between SIGLCE15 mRNA expression and the clinical characteristics of CRC patients. Twenty-three patients with transcriptome data were divided into two groups according to their status of lymph node metastasis: stages I and II (no lymph node metastasis) and stages III and IV (lymph node metastasis). Comparison of the relative expression of *SIGLEC15* (SN/NSN) between stage I and II and stage III and IV patients revealed that there were no statistically significant differences in the expression between the two groups, albeit a higher relative expression of *SIGLEC15* was observed in the later-stage patients (Figure 5A). We then divided the CRC patients into different immunological feature groups based on the status of MSI and performed comparative studies. As shown in Figure 5B, a lower relative expression of *SIGLEC15* was found in patients with MSI-H status as compared to those with microsatellite-stable (MSS) status. It has been reported that the clinical manifestation of CRC differs between patients with right-sided colorectal tumor and those with left-sided colorectal tumor (Imperial et al., 2018). We, therefore, examined whether the relative expression of *SIGLEC15* in CRC patients is associated with the primary tumor locations. It turned out that there was no major difference of relative *SIGLEC15* expression observed between the two groups (Figure 5C). However, the expression level of *SIGLEC15* only in SNs was not a critical indicator for comparison purpose, as seen among the above groups with no major differences (Supplementary Figure 4).

### Silencing *SIGLEC15* Restores the Anti-Tumor Ability of T Cells in Sentinel Lymph Nodes

**FIGURE 6:**
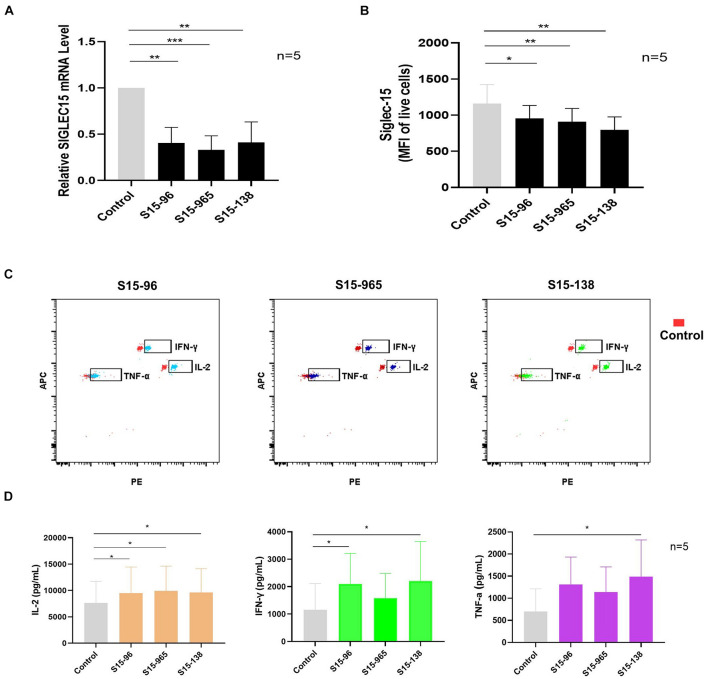
Silencing *SIGLEC15* restores the anti-tumor ability of T cells in SNs. **(A)** RT-PCR analyses of *SIGLEC15* mRNA expression in five cases of SN cells treated with control siRNA (control) or *SIGLEC15*-siRNA (S15-96, S15-965, and S15-138). **(B)** Flow cytometry analyses of Siglec-15 protein expression in five cases of SN cells treated with control siRNA (control) or *SIGLEC15*-siRNA (S15-96, S15-965, and S15-138). **(C)** Graphical representation of LEGENDplex assay of cytokine expression in control and *SIGLEC15*-siRNA-treated SN cells. **(D)** Quantification of cytokines in the supernatants 72 h after *SIGLEC15*-siRNA silencing in SN cells. *p < 0.05, **p < 0.01, and ***p < 0.001, with paired *t*-test.

To verify whether *SIGLEC15* is responsible for immunosuppression in the pre-metastatic niche, we transfected the SN cells with *SIGLEC15* siRNA. As depicted in Figure 6A, *SIGLEC15* expression was reduced in all *SIGLEC15* siRNA-expressing cell lines; among the three siRNAs, S15-138 displayed the best silencing effect. siRNA-mediated silencing of *SIGLEC15* was also validated by flow cytometry analysis (Figure 6B). We next assessed the role of *SIGLEC15* in anti-tumor activities of T cells. As illustrated in Figures 6C,D, reduced expression of *SIGLEC15* in the presence of PMA and ionomycin stimulation led to an increased release of IL-2, IFN-γ, and TNF-α in SN cells. These data indicated that *SIGLEC15* exerts an immunosuppressive effect on the pre-metastatic lymph node, and this effect can be relieved by gene silencing.

## Discussion

Sentinel lymph node is the first outpost of tumor drainage and has a unique anatomical location. SN is an important site for immune recognition and initiation of anti-tumor immunity (Chen and Mellman, 2013), while acting as a pathway for tumor metastasis and escape (Cheung and Ewald, 2016). Studies on the immune micro-environment of tumor SN would elucidate the underlying mechanisms. Increasing evidence suggests that changes in the pre-metastatic niche occur in the lymph nodes prior to the cancer cell arrival (Shriver et al., 2014; Balsat et al., 2017; Garciá-Caballero et al., 2017). So far, the molecular mechanisms involving the pre-metastatic niche, especially the lymph nodes of colorectal cancer, remain unclear. Herein, we conducted the first analysis of transcriptional regulation in the pre-metastatic niche of SN in colorectal cancer.

In this study, we identified 16 DEGs between SN and the paired NSN by high-throughput RNA sequencing and bioinformatics analysis, which comprise nine significantly upregulated genes and seven downregulated genes. Among the upregulated genes, *PLA2G2D* and *SIGLEC15* had a putative function in immunosuppression or promoting tumor growth and metastasis. While *PLA2G2D* is mainly expressed in macrophage and dendritic cells, and functions in reducing Th1 and Th17 immune responses (Miki et al., 2013, 2016), *SIGLEC15* is identified as the most upregulated gene in SN and can suppress T-cell responses (Wang et al., 2019). In addition, our study also identified *NR2F1*, *DNAJB4*, and *STON2* as the downregulated genes in SN, which could suppress tumor growth and metastasis. In these cases, while *NR2F1* is associated with cancer cell dormancy (Sosa et al., 2015), *DNAJB4* acts as a suppressor for cancer cell metastasis (Miao et al., 2018) and *STON2* negatively modulates tumor stemness and tumorigenesis (Xu et al., 2018). Moreover, GSEA identified HALLMARK_MYC_TARGETS_V2 pathway as the most significantly enriched pathway in SN. It has been reported that HALLMARK_MYC_TARGETS_V2 pathway plays a crucial role in tumor genesis and progression (Dang, 2012). The pre-metastatic SN appears to provide a micro-environment for tumor cell proliferation. Comparatively, transforming growth factor beta (TGF-β) signaling and TNF-α/nuclear factor kappa-light-chain-enhancer of activated B cells (NF-κB) signaling were more enriched in the NSN group, suggesting that the NSN may possess a relatively complete immune function. Although the roles of TGF-β in cancer are paradoxical, TGF-β signaling can inhibit cell proliferation and promote differentiation and apoptosis of colonic epithelial cells (Massagué et al., 2005). Disruption of TGF-β signaling in the colon prompts tumor progression *via* epithelial cell transformation as well as tumor–stromal interactions (Itatani et al., 2019). The transient activation of TNF-α/NF-κB signaling could contribute to the response to stimulation of the inflammatory microenvironment by cytokines (Batlle and Massagué, 2019). In the present study, alterations in the genes and gene sets could lead to the formation of an immunosuppressive niche in SN, ultimately providing bases for tumor cell colonization.

*SIGLEC15* has been shown to be a novel target for normalized cancer immunotherapy (Wang et al., 2019; Sun et al., 2021). Our study identified *SIGLEC15* as a significantly upregulated gene in SN, as verified by both RT-PCR and RNAscope assays. The RNAscope assay revealed that *SIGLEC15* was partially co-expressed with *CD163*, while *SIGLEC15* was ubiquitously distributed in lymph nodes. In order to better understand the expression of *SIGLEC15* in lymph nodes, we further analyzed the expression profiles in immune cell subsets by flow cytometry. Contrary to the previous study (Takamiya et al., 2013), we found that Siglec-15 was not exclusively expressed in M2 macrophages but expressed in other myeloid cell subsets as well. This observation may be explained by the inconsistency between the gene and protein expression levels. In line with the previous finding, we observed that Siglec-15 was significantly highly expressed in M2 macrophages compared with other cell subsets. To date, there have been relatively few studies focused on the transcriptome analysis of lymph nodes in colorectal cancer. In the study, we also analyzed the public dataset (GSE141174) from the gene expression omnibus (GEO) database, which contains the transcriptome data of lymph nodes from five colorectal patients as well as those of normal lymph nodes from non-tumor patients. But, this analysis failed to detect the difference of *SIGLEC15* expression between the two groups (Supplementary Figure 5).

It has been reported that *SIGLEC15* overexpression is correlated with favorable or unfavorable outcomes in different types and subtypes of cancer (Li B. et al., 2020). Kaplan–Meier analysis showed that *SIGLEC15* mRNA level has no significant influence in rectum adenocarcinoma (*P* = 0.27) (Li Q. T. et al., 2020). In our study, we examined whether *SIGLEC15* expression is related to clinical characteristics in CRC patients and found no correlation of *SIGLEC15* expression in SNs with clinical stage, tumor MSI status, or tumor location. Taking into account the heterogeneity of individual patients, we calculated the relative expression of *SIGLEC15* to eliminate the tissue background signal. The data showed that the relative expression of *SIGLEC15* was increased in the MSS group compared with the MSI-H group, suggesting that *SIGLEC15* may reflect immunological characteristics of colorectal cancer. Notably, there was no significant difference in the relative expression of *SIGLEC15* between later-stage CRC patients and early-stage patients, albeit the expression was slightly increased in the later-stage cases. This observation may result from a small sample size in the study. In addition, we followed up stage II patients and found no differences in the relative expression of *SIGLEC15* between patients with different disease progression (Supplementary Table 2 and Supplementary Figure 7). Therefore, it remains to be determined whether the relative expression of *SIGLEC15* in lymph nodes can serve as a prognostic indicator of a CRC patient.

While Siglec-15 has been identified as a promising target in sequence to PD-L1 in cancer immunotherapy (Wang et al., 2019), clinical development of a Siglec-15 antibody for solid tumor has been initiated (Sun et al., 2021). We also investigated whether suppressing *SIGLEC15* expression in SNs could enhance the anti-tumor function of T cells. Strikingly, siRNA-mediated silencing of *SIGLEC15* in SN increased the secretion of anti-tumor cytokines, including IL2, IFN-γ, and TNF-α, suggesting that inhibiting *SIGLEC15* expression in SNs could restore T cell function. We previously used SN T cells for adoptive cell therapy (Zhen et al., 2015). These findings indicate that *SIGLEC15* expression would benefit the effect of adoptive cellular therapy.

The study revealed that a group of genes associated with tumor immunity were highly expressed in the pre-metastatic SNs. Particularly, we showed that *SIGLEC15* is a key immune suppressor in the pre-metastatic lymph node, which might be a critical factor in the lymph node metastasis of colorectal cancer. In addition, this study suggested that *SIGLEC15* would be a new immune therapeutic target for harnessing T cells in tumor lymph nodes.

## Data Availability Statement

The data presented in the study are deposited in the NCBI SRA repository, accession number is PRJNA759656.

## Ethics Statement

The studies involving human participants were reviewed and approved by the Medical Ethics Committee of the Affiliated Hospital of Guizhou Medical University. The patients/participants provided their written informed consent to participate in this study.

## Author Contributions

HD and JT performed the experiments and contributed to data analysis and manuscript preparation. XL and RZ acquired patient data and data analysis. XW and LW performed flow cytometry and data analysis. PH conceived of the study and contributed to data analysis. YY formulated experimental design, performed data analysis, and interpreted results. All authors read and approved the final manuscript.

## Conflict of Interest

LW and PH were employed by company Sinorda Biotechnology Co., Ltd. The remaining authors declare that the research was conducted in the absence of any commercial or financial relationships that could be construed as a potential conflict of interest.

## Publisher’s Note

All claims expressed in this article are solely those of the authors and do not necessarily represent those of their affiliated organizations, or those of the publisher, the editors and the reviewers. Any product that may be evaluated in this article, or claim that may be made by its manufacturer, is not guaranteed or endorsed by the publisher.
