# Supplementary figures and images for: Siglec-15 Is an Immune Suppressor and Potential Target for Immunotherapy in the Pre-Metastatic Lymph Node of Colorectal Cancer
Source: Front Cell Dev Biol. 2021 Oct 13;9:691937. doi: 10.3389/fcell.2021.691937 (PMC8548766; doi:10.3389/fcell.2021.691937)

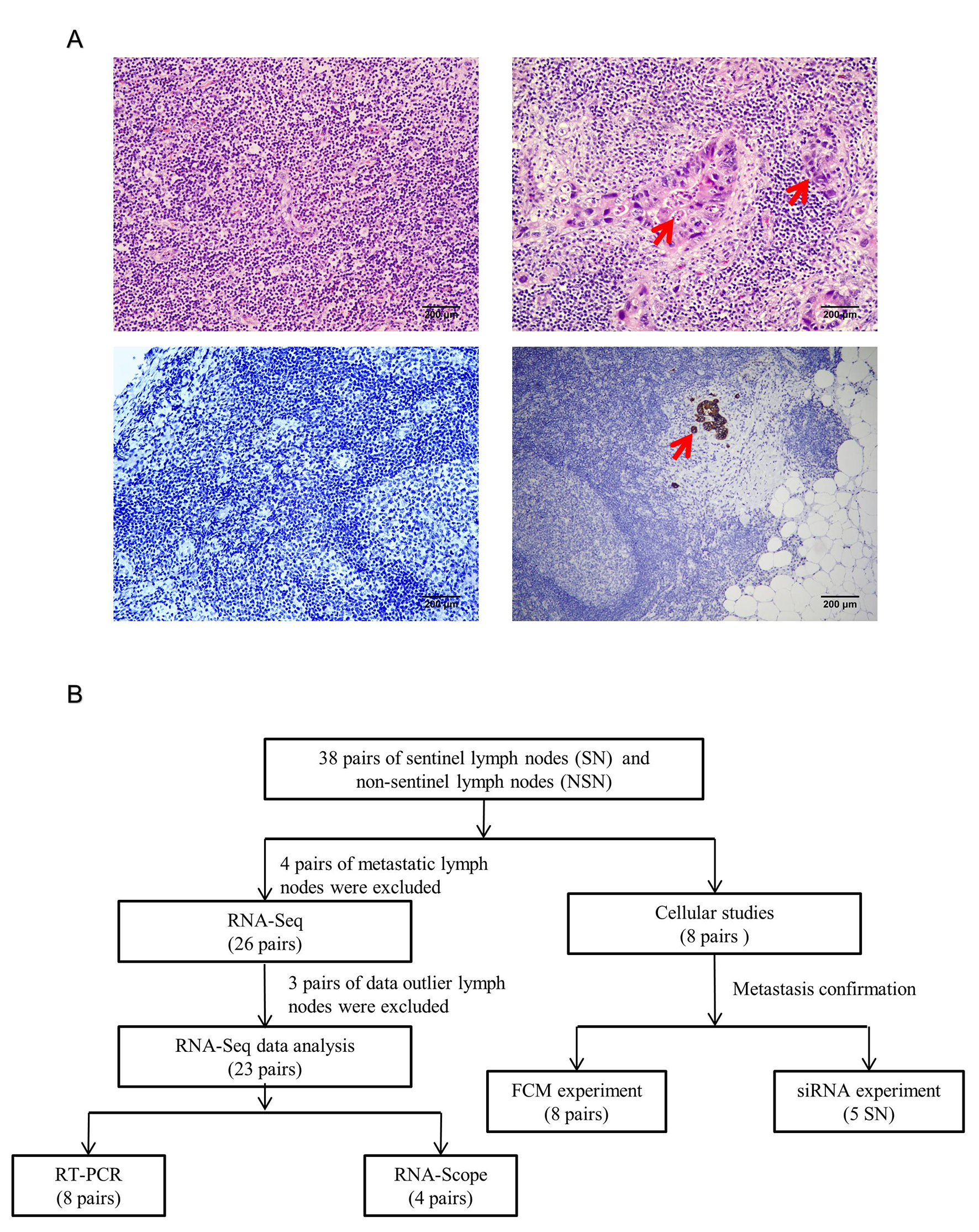

Supplement: Supplementary file 1 [file Image_1.TIF]

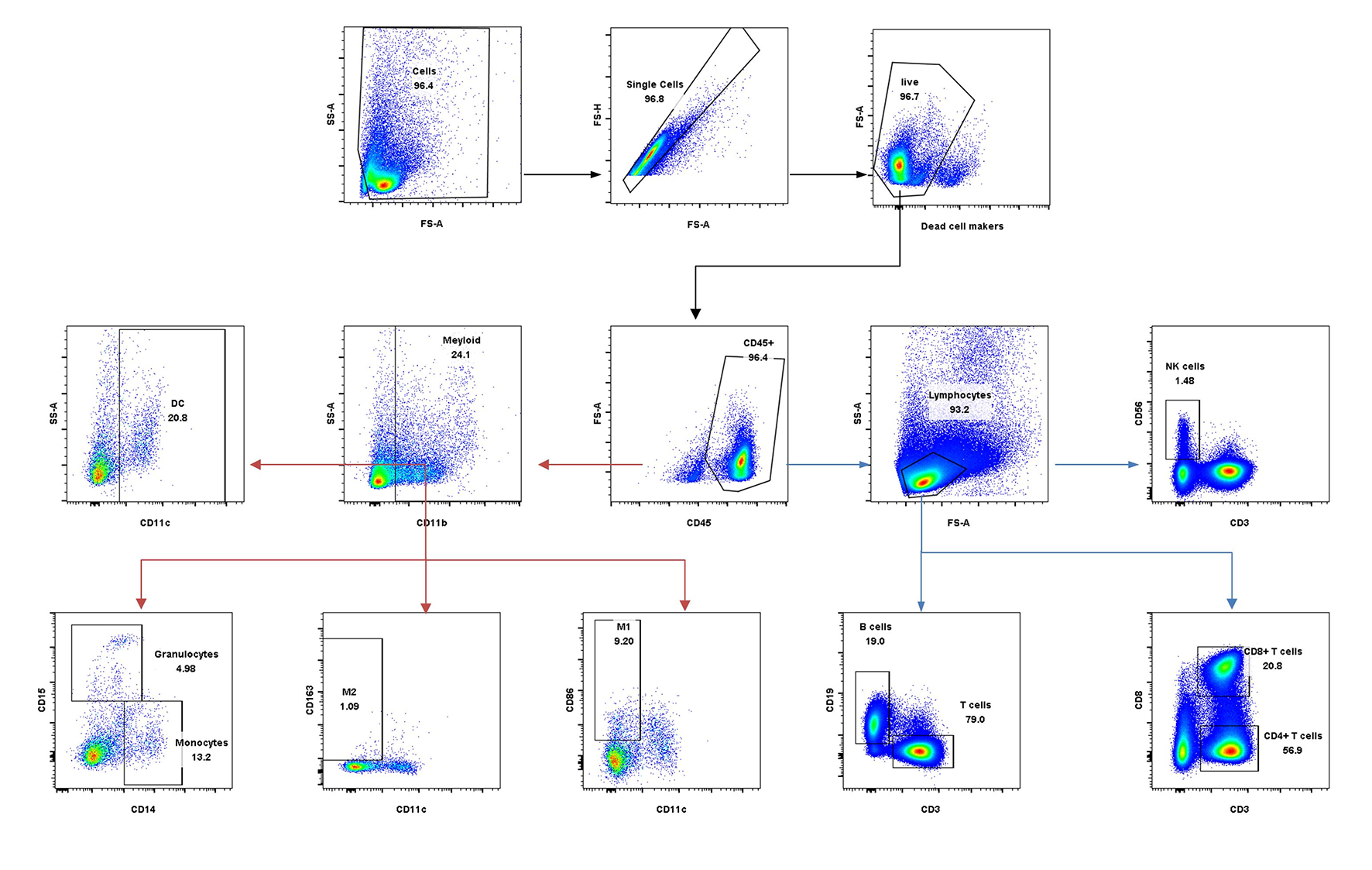

Supplement: Supplementary file 2 [file Image_2.TIF]

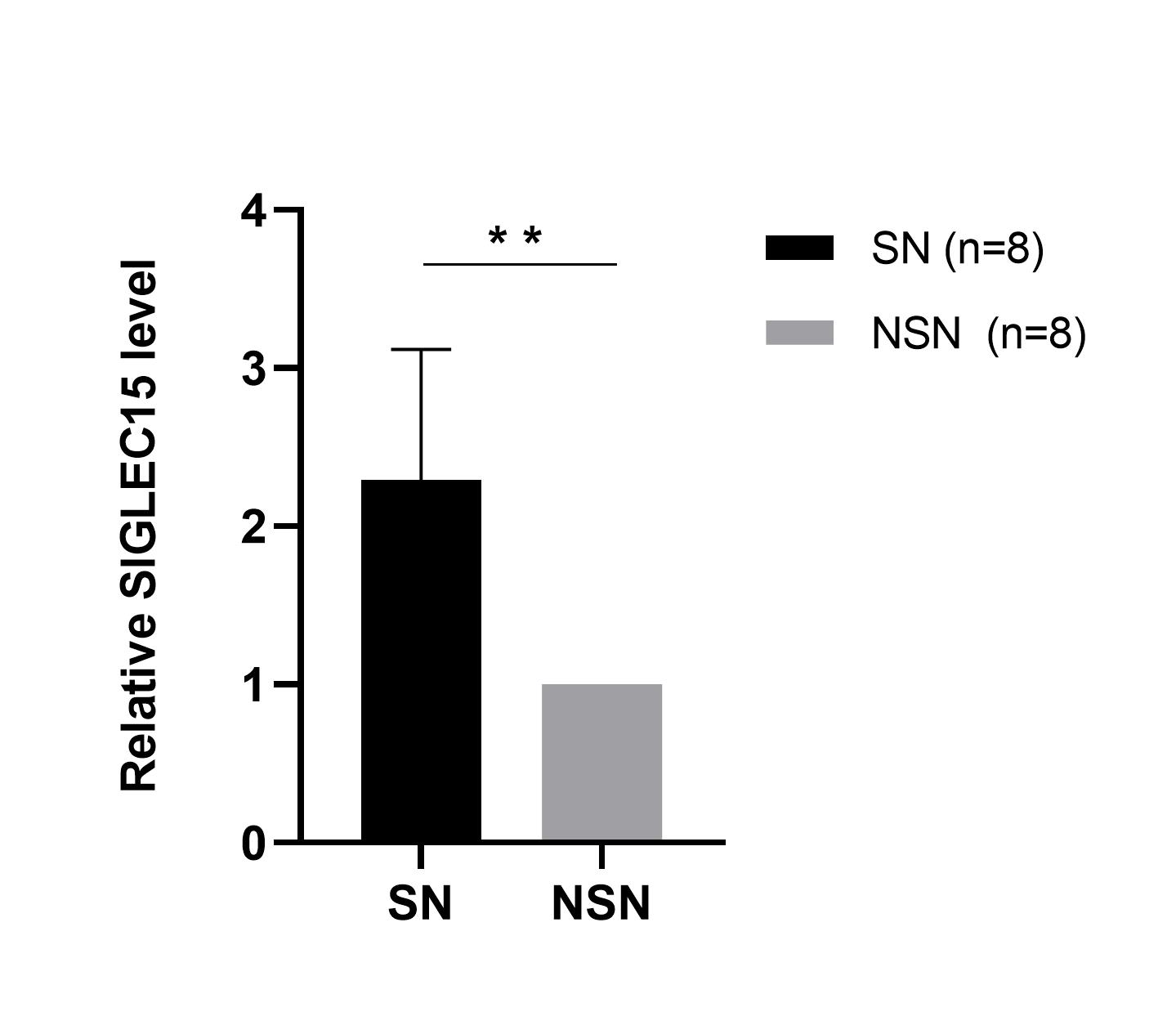

Supplement: Supplementary file 3 [file Image_3.TIF]

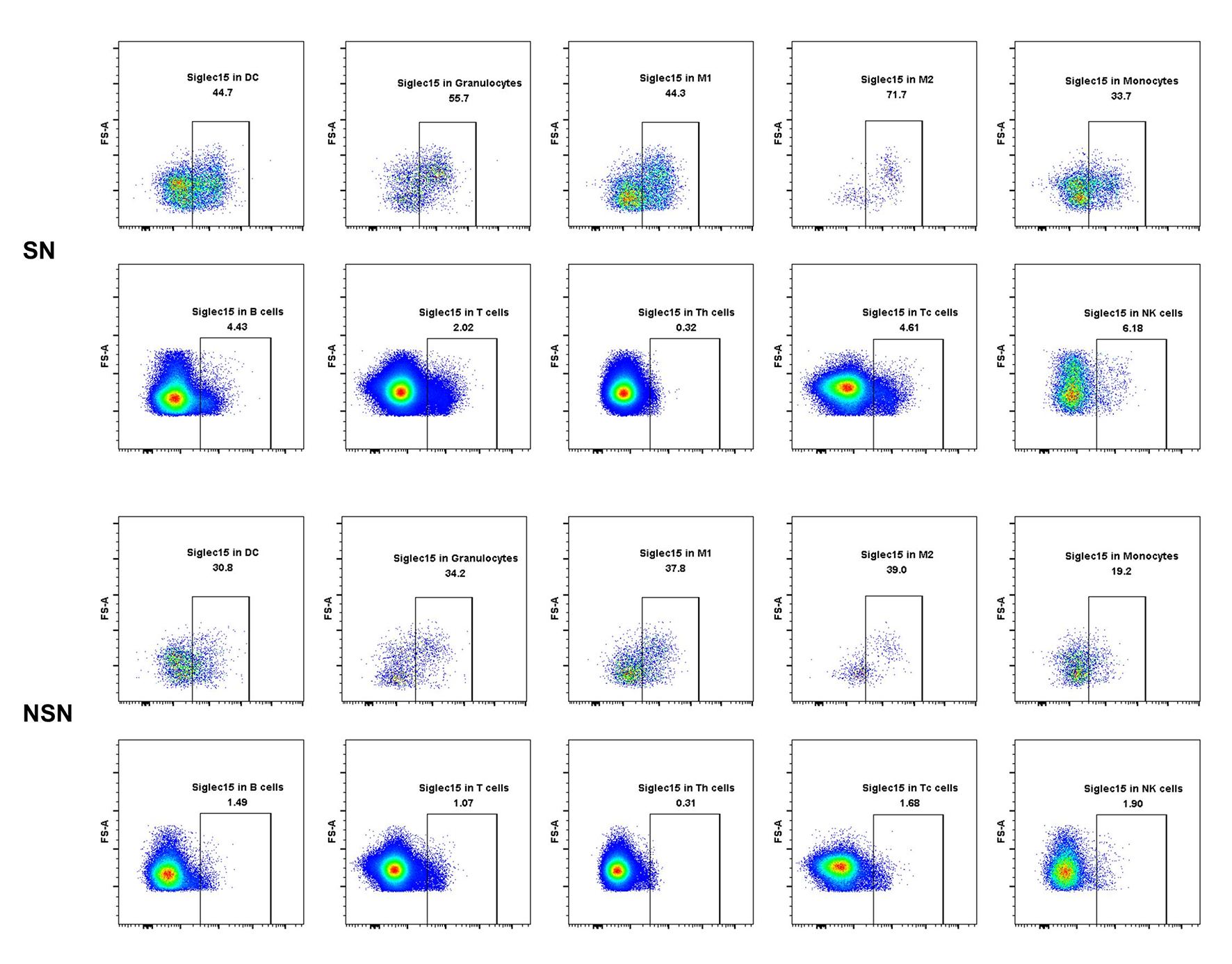

Supplement: Supplementary file 4 [file Image_4.TIF]

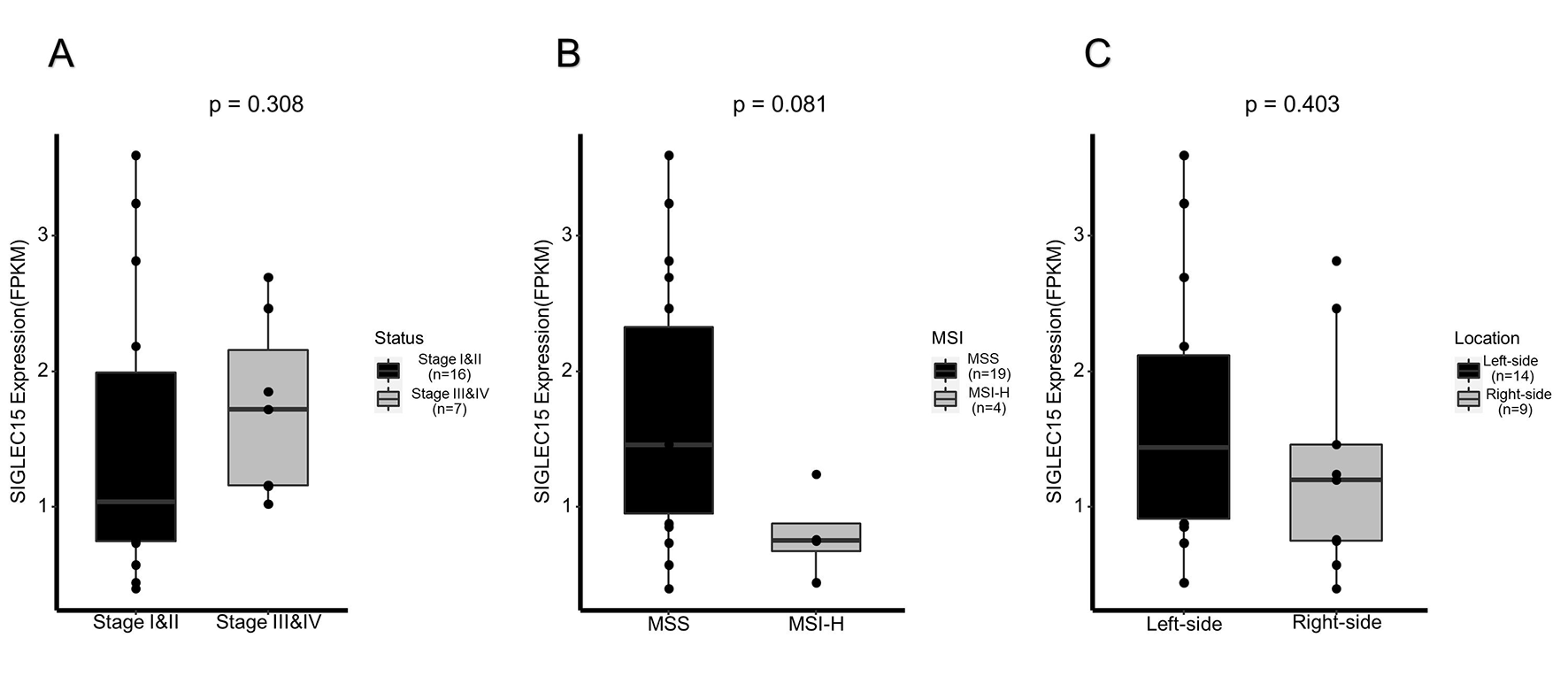

Supplement: Supplementary file 5 [file Image_5.TIF]

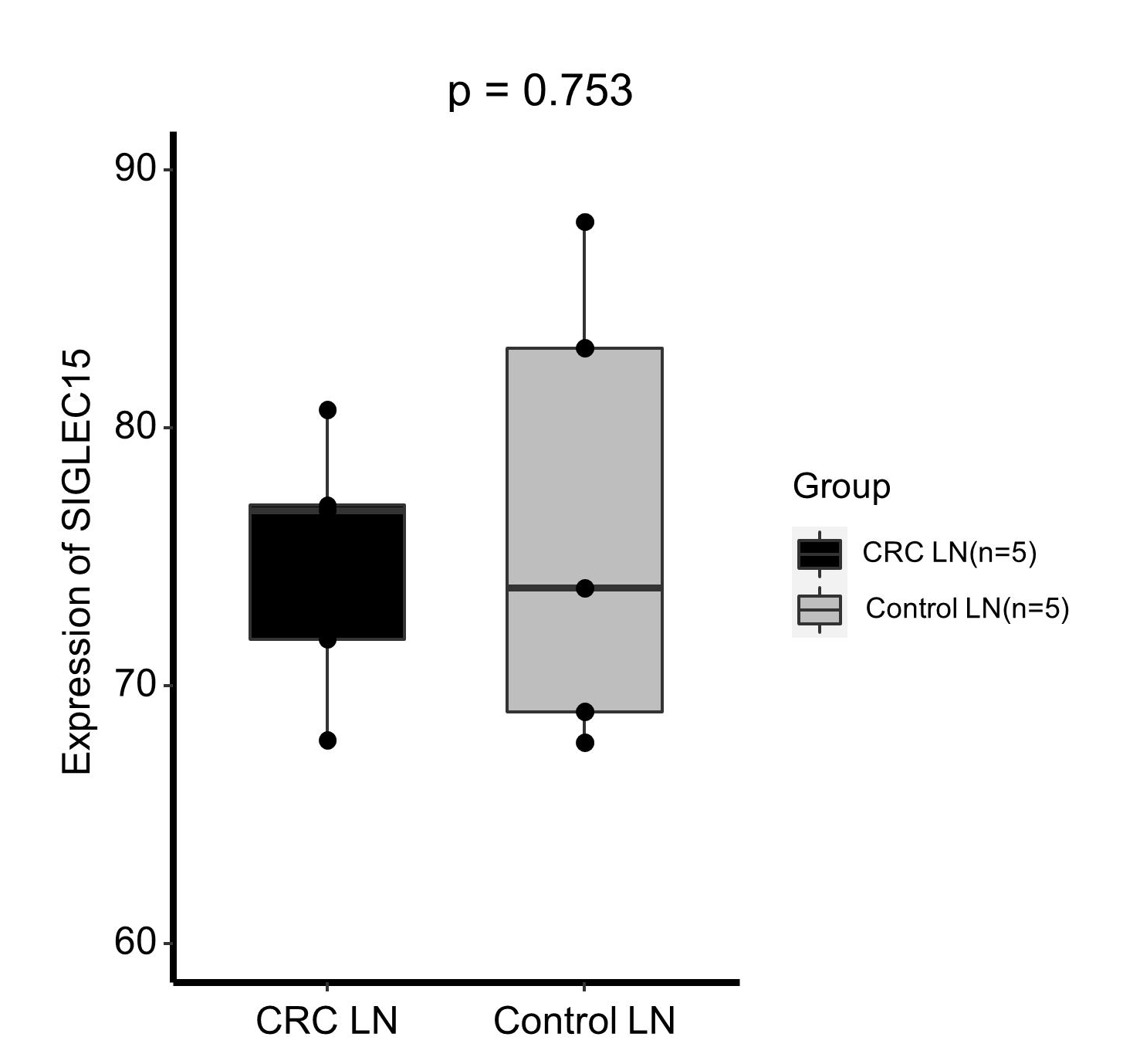

Supplement: Supplementary file 6 [file Image_6.TIF]

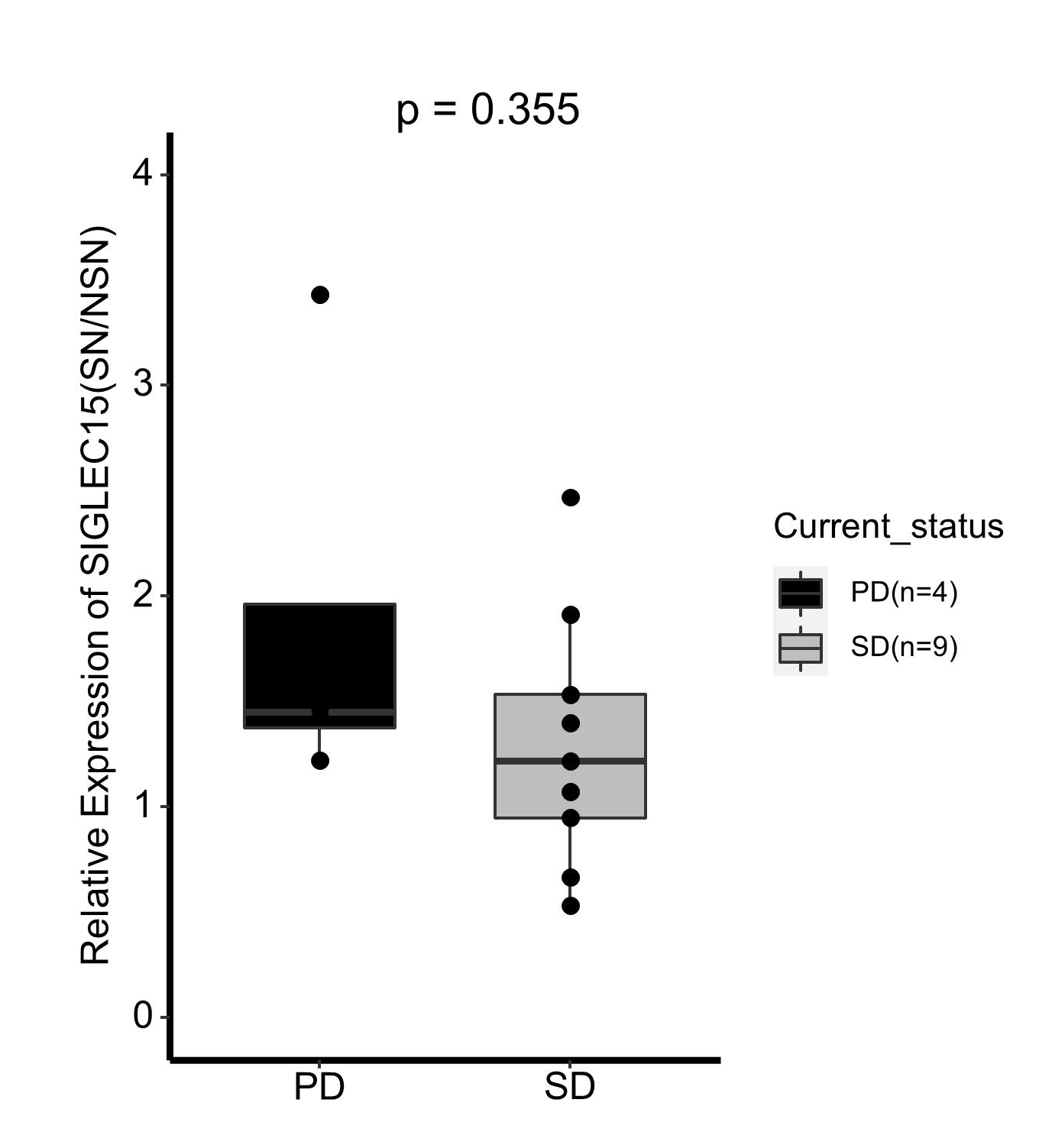

Supplement: Supplementary file 7 [file Image_7.TIF]
