# Supplementary material for: Siglec-15 Is an Immune Suppressor and Potential Target for Immunotherapy in the Pre-Metastatic Lymph Node of Colorectal Cancer
Source: Front Cell Dev Biol. 2021 Oct 13;9:691937. doi: 10.3389/fcell.2021.691937 (PMC8548766; doi:10.3389/fcell.2021.691937)
Supplement: Supplementary file 8 [file Table_1.DOCX]

**Table S1. Antibodies/reagents used for the flow cytometery assay**

| **Marker** | **Clone** | **Supplier** |
| --- | --- | --- |
| [CD45](https://www.biolegend.com/en-us/search-results/fitc-anti-human-cd45-19889) FITC | [HI30](https://www.biolegend.com/en-us/search-results?Clone=HI30) | Biolegend |
| CD86 PE | HA5.2B7 | Beckman |
| CD3 ECD | UCHT1 | Beckman |
| CD11c [PE/Dazzle™ 594](https://www.biolegend.com/en-us/search-results/pe-dazzle-594-anti-human-cd11c-antibody-10638) | 3.9 | Biolegend |
| CD163 PerCP/Cyanine5.5 | RM3/1 | Biolegend |
| CD19 PerCP/Cyanine5.5 | [HIB19](https://www.biolegend.com/en-us/search-results?Clone=HIB19) | Biolegend |
| CD15 PE-Cy7 | W6D3 | Biolegend |
| Siglec-15 | / | bio-techne |
| Donkey Anti-rabbit IgG/APC antibody | / | Absin |
| CD11b APC/Cyanine7 | ICRF44 | Biolegend |
| CD8 APC/Cyanine7 | SK1 | Biolegend |
| CD14 Pacific Blue | 63D3 | Biolegend |
| CD56 Pacific Blue | 5.1H11 | Biolegend |
| Zombie Yellow | / | Biolegend |
| Fc Receptor Blocking Solution | / | Biolegend |
