# Supplementary material for: Siglec-15 Is an Immune Suppressor and Potential Target for Immunotherapy in the Pre-Metastatic Lymph Node of Colorectal Cancer
Source: Front Cell Dev Biol. 2021 Oct 13;9:691937. doi: 10.3389/fcell.2021.691937 (PMC8548766; doi:10.3389/fcell.2021.691937)
Supplement: Supplementary file 9 [file Table_2.DOCX]

**Table S2. The follow-up information of CRC patients (N=38)**

| **Patient ID.** | **Age/sex** | **AJCC stage** | **TNM classification at surgery** | **Date** enrolled | **P**rogression free survival | **Status**§ |
| --- | --- | --- | --- | --- | --- | --- |
| Patient 1 | 69/M | Ⅳ A | T4bN1aM1a | 2018-10-25 | 7 | Dead |
| Patient 2 | 62/F | Ⅲ B | T3N1aM0 | 2018-10-30 | 11 | PD |
| Patient 3 | 50/F | Ⅲ B | T3N1aM0 | 2018-11-1 | 33 | SD |
| Patient 4 | 61/M | Ⅱ A | T3N0M0 | 2018-11-5 | / | NA |
| Patient 5 | 54/M | Ⅱ A | T3N0M0 | 2018-10-25 | 12 | PD |
| Patient 6 | 63/F | Ⅰ | T2N0M0 | 2018-11-8 | 33 | SD |
| Patient 7 | 40/M | Ⅲ B | T3N1bM0 | 2018-11-16 | 4 | PD |
| Patient 8 | 53/M | Ⅱ A | T3N0M0 | 2018-11-21 | 33 | SD |
| Patient 9 | 46/M | Ⅱ A | T3N0M0 | 2018-11-28 | 33 | SD |
| Patient 10 | 64/M | Ⅲ B | T3N1aM0 | 2018-11-28 | 12 | PD |
| Patient 11 | 62/F | Ⅱ A | T3N0M0 | 2018-11-29 | 33 | SD |
| Patient 12 | 57/F | Ⅳ A | T3N0M1a | 2018-11-29 | 30 | PD |
| Patient 13 | 74/M | Ⅱ A | T3N0M0 | 2018-12-6 | 32 | SD |
| Patient 14 | 58/M | Ⅱ A | T3N0M0 | 2018-12-7 | 32 | SD |
| Patient 15 | 66/F | Ⅰ | T2N0M0 | 2018-11-19 | 33 | SD |
| Patient 16 | 57/M | Ⅲ B | T3N2aM0 | 2018-11-27 | 4 | PD |
| Patient 17 | 73/M | Ⅲ C | T3N2bM0 | 2018-12-12 | 8 | Dead |
| Patient 18 | 76/F | Ⅰ | T2N0M0 | 2018-12-13 | 32 | SD |
| Patient 19 | 76/M | Ⅲ B | T3N1cM0 | 2018-12-10 | / | NA |
| Patient 20 | 43/M | Ⅱ A | T3N0M0 | 2018-12-14 | 32 | SD |
| Patient 21 | 42/M | Ⅱ A | T3N0M0 | 2018-12-14 | 32 | SD |
| Patient 22 | 68/M | Ⅱ A | T3N0M0 | 2018-12-12 | 32 | PD |
| Patient 23 | 63/M | Ⅳ B | T3N1cM1b | 2018-11-27 | / | NA |
| Patient 24 | 64/F | Ⅱ A | T3N0M0 | 2018-12-11 | 32 | SD |
| Patient 25 | 70/F | Ⅲ B | T3N2aM0 | 2018-12-8 | 8 | PD |
| Patient 26 | 77/F | Ⅱ A | T3N0M0 | 2018-12-12 | 8 | Dead |
| Patient 27 | 82/M | Ⅳ C | T3N1cM1c | 2018-12-13 | / | NA |
| Patient 28 | 66/M | Ⅱ A | T3N0M0 | 2018-12-21 | 20 | PD |
| Patient 29 | 46/M | Ⅱ A | T3N0M0 | 2018-12-17 | 32 | SD |
| Patient 30 | 77/M | Ⅱ A | T3N0M0 | 2018-12-23 | 32 | SD |
| Patient 31 | 66/F | Ⅲ B | T3N1bM0 | 2020-5-13 | 15 | SD |
| Patient 32 | 34/M | Ⅱ A | T3N0M0 | 2020-5-15 | 15 | SD |
| Patient 33 | 72/F | Ⅲ B | T3N1cM0 | 2020-5-20 | 15 | SD |
| Patient 34 | 78/M | Ⅱ A | T3N0M0 | 2020-5-28 | 15 | SD |
| Patient 35 | 90/F | Ⅲ B | T3N1aM0 | 2020-6-1 | 14 | SD |
| Patient 36 | 63/F | Ⅱ A | T3N0M0 | 2020-6-3 | 14 | SD |
| Patient 37 | 70/F | Ⅲ B | T3N1bM0 | 2020-6-10 | 14 | SD |
| Patient 38 | 81/F | Ⅱ A | T3N0M0 | 2020-6-11 | 14 | SD |

**§** The last contact date was 9^th^, Aug.,2021

* Patients who have transcriptome data are highlighted.

Stable disease (SD), [progression](C:/Program%20Files%20(x86)/Youdao/Dict/8.9.6.0/resultui/html/index.html#/javascript:;)  [disease](C:/Program%20Files%20(x86)/Youdao/Dict/8.9.6.0/resultui/html/index.html#/javascript:;) (PD)
